# Sonication-mediated modulation of macronutrient structure and digestibility in chickpea

**DOI:** 10.1016/j.ultsonch.2024.106904

**Published:** 2024-05-09

**Authors:** Weiyan Xiong, Gaurav Kumar, Bin Zhang, Sushil Dhital

**Affiliations:** a Bioresource Processing Research Institute of Australia (BioPRIA), Department of Chemical and Biological Engineering, Monash University, Clayton, VIC 3800, Australia; b School of Food Science and Engineering, Overseas Expertise Introduction Centre for Discipline Innovation of Food Nutrition and Human Health, South China University of Technology, Guangzhou 510640, China

**Keywords:** Ultrasound, Intact cell, Cell wall, Starch, Protein, Digestion

## Abstract

Ultrasound processing is an emerging green technology that has the potential for wider application in the food processing industry. While the effects of ultrasonication on isolated macromolecules such as protein and starch have been reported, the effects of physical barriers on sonication on these macro-molecules, for example inside whole seed, tissue or cotyledon cells, have mostly been overlooked. Intact chickpea cells were subjected to sonication with different ultrasound processing times, and the effects of sonication on the starch and protein structure and digestibility were studied. The digestibility of these macronutrients significantly increased with the extension of processing time, which, however was not due to the molecular degradation of starch or protein but related to damage to cell wall macro-structure with increasing sonication time, leading to enhanced enzyme accessibility. Through this study, it is demonstrated that ultrasound processing has least effect on whole food structure, for example, whole seeds but can modulate the nutrient bioavailability without changing the properties of the macronutrients in seed fractions e.g. intact cells, offering new scientific knowledge on effect of ultrasound in whole foods at various length scales.

## Highlights

• Ultrasound treatment (UT) has negligible effect on whole chickpea structure.
• UT enhances digestibility of macronutrients (MN) within intact chick pea cells (ICC)
• UT causes minimal structural changes to starch and protein within ICC.
• Change in Cell-wall structure is major cause for increased MN digestibility.

## 1 Introduction

The low-frequency high-intensity ultrasound (20–100 kHz, 10–1000 W/cm^2^) has been applied in aqueous phase food processing mainly as non-thermal technologies to aid food processing as well as to alter the food functionality [1]. Ultrasound-generated cavitation, primarily through the collapse of microscopic bubbles, produces significant energy in the form of localized heat, pressure, and shear forces. These forces increase mass transfer and could potentially replace certain thermal and/or chemical processes, making food processing more environmentally friendly and sustainable compared to conventional methods [2]. For example, the ultrasound technique has been applied to modify starch and protein functionality. Due to the acoustic cavitation, fissures and cracks are formed on the surface of the starch granules along with alteration of physiochemical properties including water solubility, gelatinization, retrogradation, pasting viscosity, swelling parameters and digestibility [3], [4], [5], [6], [7]. However, the strong evidence of molecular degradation of starch, leading to change in supra-molecular structure is not reported so far. In contrast, transient breakage of the chains and/or the modification of the side groups of amino acids are reported for proteins leading to the transient or permanent modification of the three-dimensional structure of the folded protein, and changes in the secondary structure of protein. This leads to the alteration of gelation, emulsification, foaming, and water-holding capacity of proteins [8], [9], [10], [11], [12], [13], [14]. The dense and semi-crystalline structure of granular starch thus seems to be more resistant to ultrasound treatment compared to proteins, at least in retention of molecular integrity.

Ultrasound, a powerful technology, could potentially be used to modulate the properties of starch and protein, within the intact cells of the legumes’ cotyledon. However, as the legume seeds are complex biological materials with different levels of compact cellular organization, it is difficult to assess the effects of ultrasound treatments on the individual cells. Most of the work investigated how the ultrasound affects the properties of the whole seeds scale (1–2 cm), e.g. hydration, germination, the ultrasound wave may damage the surface of the seed coat therefore accelerating the mass transfer leading to a higher hydration rate and germination rate [15], [16], [17], [18]. Regarding to the cellular level of the legumes, and the macronutrients encased in the cells both for isolated intact cells and in-planta cells scant information is available for the sonification effects. There is also limited understanding of the “cushion effect” on the cells embedded in the cotyledon, which might be shielded from the ultrasonic effects.

This study aims to elucidate the direct impact of ultrasound on cell walls and encapsulated starch and protein by isolating intact cells (100–200 µm) and subjecting them to varied durations of ultrasound treatment under controlled temperatures. Moreover, to determine whether in-planta conditions offer a protective “cushion effect” on the intact cells, intact cells were further isolated from sonicated whole bean. Individual components, i.e., starch, protein and cell wall were isolated to study their compositional, thermal, and supramolecular properties. Subsequently, effect of ultrasound and food structure (in-planta vs isolated intact cells) on the digestibility of starch and protein present within the intact chickpea cells were explored. The current work is based on “lowest” whole food model provides crucial insights into the mechanistic underpinnings of non-thermal ultrasound treatment of legume and give a new insight of the impact of ultrasound the whole foods’ structure and properties.

## 2 Methods and materials

Chickpea seeds were purchased from a local supermarket in Melbourne, Australia. Pepsin (1:2500LR) was purchased from ChemSupply, Australia, pancreatin (P7454) was obtained from Sigma-Aldrich, USA. The total starch assay kit (K-TSTA) was purchased from Megazyme, Ireland. All other chemicals were of analytical grade.

### 2.1 Isolation of intact cells

**Fig. 1:**
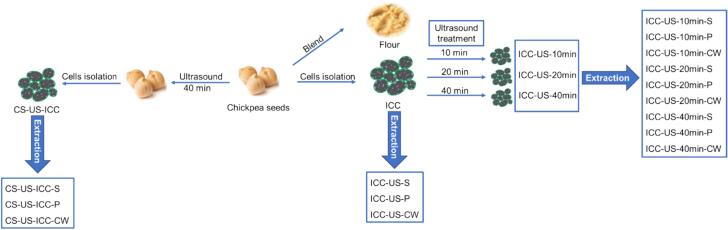
Schematic figure of the sample preparation. (ICC, untreated intact chickpea cells; ICC-US-10 min, intact chickpea cells treated with 10 min ultrasound; ICC-US-20 min, intact chickpea cells treated with 20 min ultrasound; ICC-US-40 min, intact chickpea cells treated with 40 min ultrasound; CS-US-ICC, intact chickpea cells isolated from ultrasound treated chickpea seeds; S, stands for the corresponding starch isolates, P stands for the corresponding protein isolates, CW stands for the corresponding cell wall isolates).

Intact chickpea cotyledon cells (ICC) were isolated following the method by Dhital et al (2016) with slight modifications [19]. Briefly, dried seeds were soaked in excessive water with 1 % NaHCO_3_ addition and stored in 4-degree refrigerator overnight to promote the soaking and separation process whilst limiting the in-situ enzyme activity. Then seeds were heated at 70 °C for 30 min in excess water with gently magnetic stirring to simulate the cooking process. After that, heated seeds were disintegrated with cold press juicer (Healthy Choice) and sieved through different sieve sizes. Intact chickpea cells passed through a 150 μm sieve but retained on 50 μm sieve were collect and freeze dried. Meanwhile, the cooked chickpea seeds were dehulled and were blended with a blender and freeze dried. The dried mass passed through 50 μm sieves are considered as chickpea flour (Fig. 1).

### 2.2 Ultrasound treatment

Intact chickpea cells (ICC) or whole chickpea seeds were suspended in distilled water (10 % w/w) and mixed well. The suspension was treated by a QSONICA Q500 ultrasonic processor (20 kHz, 500 W) (Qsonica LLC., Newtown, CT, United States) equipped with a titanium probe (25.4 mm diameter). The probe was immersed in the suspension (2 cm from the top). The ultrasonic treatments were conducted for different durations (10 min, 20 min and 40 min) and performed at 50 % power amplitude under pulse mode (1 s on, 1 s off) with iced-water bath to control the temperature at room temperature. The processed intact chickpea cells samples were stored under −20 ℃, freeze-dried, and labelled as ICC-US-10 min, ICC-US-20 min and ICC-US-40 min. Besides, the chickpea seeds were treated with ultrasound for 40 min and then the intact cells were isolated, this sample was named as intact chickpea cells isolated from whole seeds treated with ultrasound (CS-US-ICC). The process of samples preparation is shown in Fig. 1.

### 2.3 Isolation of starch, protein and cell wall

All chickpea cell samples were blended with a blender to break the cell wall. The broken cells slurry was sieved through a 50 μm sieve to get the mixture of starch and protein (Fig. 1). The retentate larger than 50 μm primarily consisted of cell walls. The retentate was further purified to obtain cell wall using method described by Gartaula et al (2017) with minor modifications [20]. Collected cell wall residue was incubated with 45 mL PBS containing pancreatin (0.1 g/mL) under 37 ℃ overnight to hydrolyse the starch and protein. After hydrolysis, the mixture was wet-sieved through a 50 µm screen under running water to remove smaller particulate matter. The residue was then freeze-dried to obtain cell wall samples (Fig. 1).

The protein and starch isolations were performed in line with Shrestha et. al. (2023) [21] with slight modification. The protein and starch mixture were suspended in water, pH was adjusted to 9.0 with 0.2 M NaOH and stirred at 900 rpm for 1 h at room temperature to solubilize the protein. The solution was left for 1 h to aid the sedimentation of the starch. Afterwards, the supernatant and the sediment were separated. The sediment was washed three times with water and centrifuge to recover the starch. To recover solubilised proteins the pH of the solution was adjusted to pH 4.5 with 0.2 M HCl and allowed for protein precipitation at 4 ℃ for 12 h then centrifuged (10,000g, 10 min) to recover the precipitated protein. The isolated protein was resuspended in water and the pH was adjusted to ∼ 7.0. The isolated starch and protein were freeze-dried and stored in a desiccator for further analysis.

### 2.4 Determination of starch and protein content

The total starch content of all chickpea cells and flour samples were determined with Megazyme Total Starch Assay kit (K-TSTA-100A) according to the manufacturer's instructions.

The total protein content of all chickpea cells and flour samples were determined with Dumas’s method employing a FlashSmart elemental analyser (Thermo Fisher Scientific, USA) to determine the nitrogen content. The total protein was calculated by multiplying the nitrogen content with a conversion factor of N × 6.25.

### 2.5 X-ray diffractometry

All isolated starch samples (ICC-S, CS-US-ICC-S, ICC-US-10 min-S, ICC-US-20 min-S, ICC-US-40 min-S, Fig. 1) were analysed with an X-ray diffractometer (Rigaku, Miniflex 600) operating at 40 KV and 20 mA with Cu Kα radiation (λ) at 0.154 nm. The scanning region was set from 4° to 35° of the diffraction angle 2θ. A step interval of 0.02° and a scan rate of 0.5°/min at room temperature were employed. The relative crystallinity was calculated as the ratio of the crystalline peak area to the total diffraction using the PeakFit software (Version 4.0, Systat Software Inc., San Jose, CA, USA).

### 2.6 FT-IR

FT-IR spectroscopy of the starch (ICC-S, CS-US-ICC-S, ICC-US-10 min-S, ICC-US-20 min-S, ICC-US-40 min-S, Fig. 1) and protein (ICC-P, CS-US-ICC-P, ICC-US-10 min-P, ICC-US-20 min-P, ICC-US-40 min-P, Fig. 1) samples isolated from untreated and ultrasound treated intact cell was performed on a FT-IR spectrometer (Perkin Elmer spectrum 2) fitted with a universal attenuated total reflection (UATR) accessory and a diamond crystal. The scanning region set from 4000 to 400 cm^−1^ with a resolution of 4 cm^−1^ over 32 scans [22].

### 2.7 Differential scanning calorimetry

A differential scanning calorimeter (DSC, 2500, TA company) fitted with an intra-cooler was used to examine the thermal properties. Starch (ICC-S, CS-US-ICC-S, ICC-US-10 min-S, ICC-US-20 min-S, ICC-US-40 min-S, Fig. 1) or protein (ICC-P, CS-US-ICC-P, ICC-US-10 min-P, ICC-US-20 min-P, ICC-US-40 min-P, Fig. 1) samples isolated from untreated and ultrasound treated intact cells (Fig. 1) (∼3 mg, dry starch basis) were mixed with 7 μL distilled water in a Tzero aluminium liquid DSC pan and hermetically sealed. The samples were scanned at a heating rate of 10/°C min from 30 to 110 °C. The enthalpy change (Δ*H*), onset (*To*), peak (*Tp*) and conclusion (*Tc*) temperatures were analysed within the TA Trios software.

### 2.8 SDS-PAGE

SDS-PAGE analysis was carried out according to Shrestha et al (2023) [21]. Briefly, protein isolates (ICC-P, CS-US-ICC-P, ICC-US-10 min-P, ICC-US-20 min-P, ICC-US-40 min-P) were mixed with water to make a 1 mg/mL suspension. This suspension was rotated on a tube roller at a speed of 70 rev/min for 1 h, then centrifuged at 10,000 g for 10 min. The analysis was conducted for both reducing and non-reducing conditions. For non-reducing conditions, 15 μL of the supernatant was mixed with 5 μL Laemmli buffer, whereas for the reducing conditions, 4.5 μL Laemmli buffer and 0.5 μL β-mercaptoethanol were used and then heated at 90 °C for 5 min and centrifuged at 6700 rpm for 1 min. Electrophoresis was performed using a 1x running buffer at 200 V for 40 min. Gels were stained with Coomassie Blue for 30 min and destained with Coomassie destaining solution for 24 h.

### 2.9 Monosaccharide composition

The determination of neutral sugar composition of the cell wall polysaccharides isolated from all intact cells samples (ICC-CW, CS-US-ICC-CW, ICC-US-10 min-CW, ICC-US-20 min-CW, ICC-US-40 min-CW, Fig. 1) was done following the Salvador et al (2000) [23]. Briefly, 5 mg of each cell wall sample was hydrolysed with H_2_SO_4_ in a screw-capped test tube according to the Saeman hydrolysis [24]. Then the pH of the solution was neutralised to 7.0 with 0.5 M NaOH. The high-performance anion-exchange chromatography (ICS-6000, thermos scientific, USA), using a CarboPac PA10 column (2 × 250 mm) was performed for identification and quantification of monosaccharides. Fucose, Rhamnose Arabinose, galactose, glucose, xylose and Fructose were used as standard references.

### 2.10 Total uronic acid content

Uronic acid levels were measured using a modified Blumenkrantz colorimetry method [25]. Cell wall samples (ICC-CW, CS-US-ICC-CW, ICC-US-10 min-CW, ICC-US-20 min-CW, ICC-US-40 min-CW, Fig. 1) (3 mg) were hydrolysed in concentrated sulfuric acid (1.0 mL), then diluted with distilled water to 10 mL. Calibration standards of D-galacturonic acid were prepared. Samples and standards (400 μL) were mixed with H_2_SO_4_/sodium tetraborate and heated in boiling water, followed by cooling down to the room temperature in iced-water bath. After that, 60 μL m-phenylphenol solution (0.15 % solution of *meta*-hydroxydiphenyl in 0.5 % NaOH) was added, mixed, and incubated for 15 min. Then the absorbance at 525 nm was measured, and uronic acid concentration was calculated based on the standard curve.

### 2.11 Scanning electron microscopy (SEM)

Scanning electron microscopic images of all the isolated cell samples (ICC, CS-US-ICC, ICC-US-10 min, ICC-US-20 min, ICC-US-40 min, Fig. 1), all the samples were prepared by affixing them onto a circular metal stub using double-sided adhesive carbon tape, then samples were coated with gold using a sputter coater (Quorum SC7620, United Kingdom). The images were acquired using a Phenom XL scanning electron microscope (Thermo Fisher Scientific, USA) at an accelerating voltage of 10 kV at 500×, 1800 × magnifications.

### 2.12 Confocal laser scanning microscopy (CLSM)

Confocal laser scanning microscopy was conducted following the method described by Li et al (2019) [26] with slight modification. Untreated intact chickpea cells (ICC) and intact chickpea cells treated with ultrasound for 40 min (ICC-US-40 min) (∼1 mg) was dispersed in 1 mL of FITC-dextran solution (70 kDa, 2 mg/mL) in a microcentrifuge tube and incubated at 4 ℃ in the dark overnight allowing the diffusion of probes into the cells. Then one drop of the mixture was spread onto a glass slide and observed under a confocal laser scanning microscope (FV3000, Olympus, Japan) at 100 × magnification. The excitation wavelength of the argon-ion laser was set at 488 nm and the emission light was detected from 488 to 523 nm.

### 2.13 In vitro digestion

The INFOGEST in vitro digestion method was performed with slight modification [27]. The digestion fluids of the simulated oral (SSF), gastric (SGF) and intestinal phases (SIF) were prepared following the protocol. Intact cells (ICC, CS-US-ICC, ICC-US-10 min, ICC-US-20 min, ICC-US-40 min, Fig. 1) and flour samples (80 mg) were weighed in a 50 mL falcon tube. 2 mL SSF was added and incubated at 37 ℃ for 2 min, then 4 mL SGF containing pepsin (640 units, 1:2500LR, Chemsupply, Australia) was added and incubated at 37 ℃ for 120 min to imitate gastric digestion. After that, 8 mL of SIF containing pancreatin (128 units, P7545, Sigma-Aldrich, USA) was added and incubated in the 37 ℃ water bath for 2 h to imitate the intestinal digestion.

For protein digestion, the spectrophotometric ophthaldialdehyde (OPA) method was used to quantify free amino groups (–NH2) released in the digested samples. The 0-hour sample was collected at the beginning of the gastric digestion phase. Aliquots (100 μL) were collected at each time point and added to 400 μL of TCA solution (15 % w/w) to halt the hydrolysis. To determine the total –NH2 in undigested samples, the samples were hydrolysed with 6 M HCl (5 mg protein/mL) at 110 ℃ for 24 h. Free amino groups quantified by OPA at different concentration of L-serine (500, 250, 125, 62.5, 31.25 mg/mL) were used as standard to generate the standard curve. The degree of protein hydrolysis (DH) was calculated as indicated in the following equation:

$DH=\frac{NH2\left(DS\right)-NH2\left(0\right)}{NH2\left(total\right)-NH2\left(0\right)}\times 100$where: NH_2_ (DS) = Free amino groups from digested sample.

NH_2_(0) = Free amino groups from samples at time 0 of digestion

NH_2_(total) = Total amount of NH_2_ present in undigested sample.

For starch digestion, the 0 h was collected after the gastric digestion, the aliquots (50 μL) were collected at regular time intervals and added to 950 μL Na_2_CO_3_ (0.5 M) solution to stop enzyme hydrolysis. The released reducing sugar in the supernatant was measured using the 4-hydroxybenzoic acid hydrazide (PAHBAH) assay. An aliquot of the supernatant (100 μL) was transferred into 1.0 mL freshly-prepared PAHBAH solution, followed by incubation in a water bath (100 °C) for 5 min and then cooling to room temperature. Different concentration of maltose (250, 125, 62.5, 31.25, 15.625 ug/mL) was used to draw the standard curve. The absorbance was measured at 410 nm and used for the calculation of the digested starch (%) according to the maltose equivalents released.

### 2.14 Statistical analysis

The experimental data were expressed as means with standard deviations of at least duplicate measurements. Analysis of variance (ANOVA) was used to determine the least significance at p < 0.05 with Tukey's multiple-range test using SPSS 26.0 software for Windows. All the Figures in the present study were visualised using GraphPad Prism 9.3.1.

## 3 Results and discussion

### 3.1 In vitro digestion

**Fig. 2:**
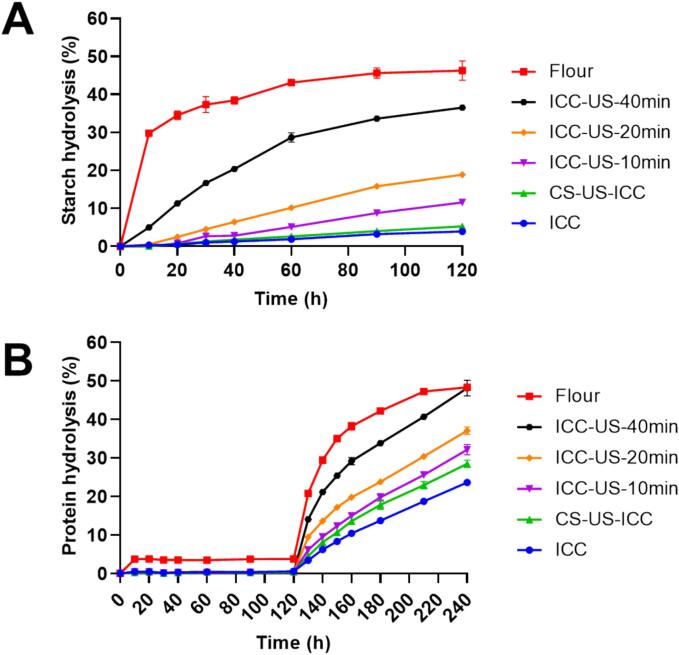
Digestogram for starch (A) and protein (B) for ultrasound-treated chickpea intact cells and chickpea flour (Flour, chickpea flour; ICC, untreated intact chickpea cells; ICC-US-10 min, intact chickpea cells treated with 10 min ultrasound; ICC-US-20 min, intact chickpea cells treated with 20 min ultrasound; ICC-US-40 min, intact chickpea cells treated with 40 min ultrasound; CS-US-ICC, intact chickpea cells isolated from ultrasound treated chickpea seeds.).

Fig. 2 demonstrates the *in-vitro* gastrointestinal digestion of starch and protein of chickpea cells or chickpea flour. As shown in Fig. 2A, the flour (cooked-blended chickpea seeds, freeze-dried), which already lost the cell structure, has the highest rate and extent of digestion, as 30 % of starch was digested within the first 10 min, which increased to 46.28 % after 120 min. Compared with the starch of flour, sample starches within the cell wall structure (intact cells) have lower and steady digestion rates. More specifically for the intact chickpea cells without the ultrasound treatment (ICC), only 3.94 % of starch was digested after 120 min of small intestinal digestion, confirming that the intact cell wall structure acted as a strong barrier to protect the inner macronutrients [19], [28]. However, with the prolongation of ultrasound treatment time, more starch was digested during the whole digestion process. With an increase in ultrasound processing time from 10 min to 40 min, the starch digestibility at the end of the digestion increased from 11.58 % to 36.52 % (Fig. 2A), suggesting the ultrasound treatment can upregulate the digestion of starch entrapped in the cell wall. In the case of cells isolated from ultrasound-treated whole chickpea seeds sample (CS-US-ICC), no significant difference was observed in starch digestion compared to the non-ultrasound intact chickpea cells sample (ICC) (Fig. 2A), suggesting that during the 40 min ultrasound treatment, the collapse of the cavitation bubbles which generated a micro-jet was contributing to the damage of the surface of the grain however has limited ability to penetrate the entire seed grain and does not seem sufficient to impact starch digestion [18], [29], [30].

Similarly, the effect of ultrasound on protein hydrolysis was similar to that of starch digestion (Fig. 2B). The chickpea flour (blended cooked chickpea seeds, freeze dried) displays a faster and higher extent of hydrolysis (48.32 % at the end of digestion process) compared to the intact cell samples throughout the whole digestion process (Fig. 2B). During the gastric digestion phase, no significant difference is observed among the intact cell samples. In contrast, from the beginning of the small intestinal phase, all ultrasound samples demonstrate higher protein hydrolysis compared to the untreated intact cells (ICC). This shows the synergy among starch and protein hydrolysis as both retains as matrix inside the cotyledon cells. However, for the cells isolated from ultrasound-treated whole chickpea seeds (CS-US-ICC), only protein digestion was only 5 % higher than ICC (Fig. 2B), suggesting the ultrasound treatment has minor effect on changing the structure of whole chickpea therefore leading to the nominal increase of entrapped protein digestibility compared to direct ultrasound treatment of isolated cells.

In contrast to the starch digestion, protein has higher hydrolysis rates, this may be attributed to the lower molecular weight of proteolytic enzymes, approximately 24–35 kDa, compared to pancreatic α-amylase, which is about 50–60 kDa [31], [32], allowing the faster diffusion inside the porous cells and protein −starch matrix.

The observed enhancement in both starch and protein hydrolysis with prolonged ultrasound treatment indicates that ultrasound can effectively modify the digestibility of macronutrients entrapped within legume cells when the ultrasound was carried on a smaller scale (e.g. intact cell). This alteration in digestibility may arise from both changes in the structural properties of starch and protein caused by ultrasound treatment, and/or from the acoustic waves making the surface of the intact legume cells more porous. However, the latter (change in cell structure) has a more profound effect, as discussed in the succeeding sections.

### 3.2 Effects of ultrasound treatment on the structural characteristics of starch

In order to investigate the effects of ultrasound treatment on the structural characteristics of starch inside of intact chickpea cells, the starch was isolated from all isolated cells samples (ICC-S, CS-US-ICC-S, ICC-US-10 min-S, ICC-US-20 min-S, ICC-US-40 min-S, Fig. 1) and the thermal properties, FTIR spectrum, XRD pattern of the isolated starch were studied.

#### 3.2.1 X-ray diffraction pattern of isolated starch

**Fig. 3:**
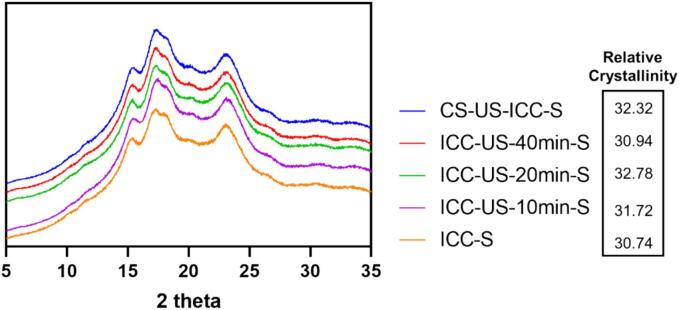
X-ray diffractograms of ultrasound treated chickpea starch isolates (ICC-S, starch isolated from intact chickpea cells; ICC-US-10 min-S, starch isolated from intact chickpea cells treated with 10 min ultrasound; ICC-US-20 min-S, starch isolated from intact chickpea cells treated with 20 min ultrasound; ICC-US-40 min-S, starch isolated from intact chickpea cells treated with 40 min ultrasound; CS-US-ICC-S, starch isolated from intact chickpea cells obtained from ultrasound treated chickpea seeds.).

Fig. 3 presents the X-ray diffraction patterns of the starch isolated from intact chickpea cells before and after sonification. All of the starch samples exhibited C-type starch XRD pattern with major peaks at 15.2°, 17.2°, 18.1°, and 23.4° 2θ [33]. The signature peak at 5.3° 2θ was not observed as chickpea starch is known to have less proportion of B-unit cells compared to other legumes (e.g. pinto bean starches) [34]. The freeze-drying operation used for drying the starch/cells is known to affect the 5.3° 2θ signature peak compared to other peaks in B polymorphic starches [35]. The relative crystallinity of starch isolated from intact cells treated with ultrasound for 0 (ICC-S), 10 (ICC-US-10 min-S), 20 (ICC-US-20 min-S), and 40 min (ICC-US-40 min-S), and starch isolated from intact cells of chickpea seeds treated with 40 min of ultrasound (CS-US-ICC-S) were 30.74 %, 31.72 %, 32.78 %, 30.94 % and 32.32 %, respectively. Prior research suggests that ultrasound waves may firstly disrupt the starch's amorphous regions which are more vulnerable, causing the crystalline regions which are more resistant become more prominent, and leading to the initial increase in relative crystallinity. With extended ultrasound treatment, the crystalline regions might also suffer damage from the ultrasound energy, leading to a decrease in relative crystallinity [3], [6], [36]. The starch isolates of the intact cells obtained from ultrasound-treated chickpea seeds has higher relative crystallinity compared to the starch isolated from the 40 min ultrasound-treated intact cells, possibly because the intact grain structure shields the starch from the direct effects of the ultrasound waves.

#### 3.2.2 Fourier transform infrared (FT-IR) spectroscopy analysis

**Fig. 4:**
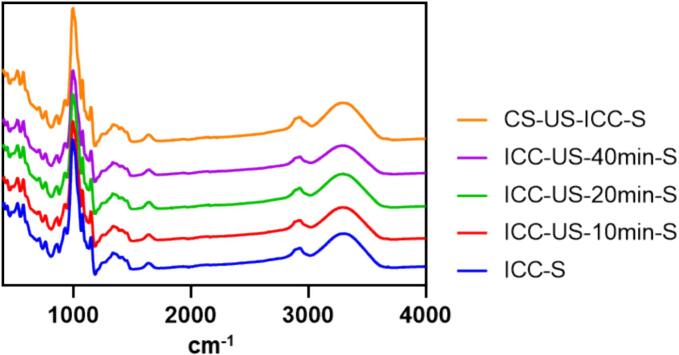
FT-IR spectra of ultrasound treated chickpea starch isolates (ICC-S, starch isolated from intact chickpea cells; ICC-US-10 min-S, starch isolated from intact chickpea cells treated with 10 min ultrasound; ICC-US-20 min-S, starch isolated from intact chickpea cells treated with 20 min ultrasound; ICC-US-40 min-S, starch isolated from intact chickpea cells treated with 40 min ultrasound; CS-US-ICC-S, starch isolated from intact chickpea cells obtained from ultrasound treated chickpea seeds.).

The FT-IR spectra of isolated starch samples (ICC-S, CS-US-ICC-S, ICC-US-10 min-S, ICC-US-20 min-S, ICC-US-40 min-S, Fig. 1) are depicted in Fig. 4, where all starch samples exhibit similar FT-IR spectra. A broad band around 3000–3600 cm^−1^ is observed, this region is often associated with O-H stretching vibrations indicating the presence of hydroxyl groups. The peak near 2900 cm^−1^ corresponds to C-H stretching vibrations. Major bands characteristic of starch, found in the 900–1200 cm^−1^ region, are related to C-O and C-C stretching vibration modes, with notable bands centred at 995, 1022 and 1047 cm^−1^. The absorbance ratios of 1022/995 cm^−1^ and 1047/1022 cm^−1^ have been widely used to characterise the starch structure. The 1047/1022 cm^−1^ ratio is often used to assess the relative proportion of crystalline to amorphous regions within starch. A higher 1047/1022 cm^−1^ ratio suggests a greater proportion of ordered, crystalline structures within the starch. Conversely, the amorphous extent of the short-range ordered structure of starch is positively correlated with the 1022/995 cm^−1^ ratio [37]. The ratios of 1047/1022 cm^−1^ and 1022/995 cm^−1^ are listed in Table 1. The ratio of 1047/1022 cm^−1^ gradually increases from 1.95 (ICC-S) to 2.24 (ICC-US-20 min-S) and then decreases slightly to 2.14 (ICC-US-40 min-S). The ratio of 1022/995 cm^−1^ remained relatively stable, with minor fluctuations from 0.40 (ICC-S) to 0.42 (ICC-US-40 min-S). These results align with the XRD findings, suggesting that ultrasound treatment initially enhances the crystalline structure of starch. With prolonged ultrasound treatment, however, the crystalline structure may be disrupted. Nonetheless, the changes in the starch crystalline structure appear to be minor.

**Table 1:** Short-range ordered structure ultrasound treated chickpea starch isolates.^1^

Samples^2^	Absorbance ratio	
	1047/1022 cm^−1^	1022/995 cm^−1^
ICC-S	1.95 ± 0.06a	0.40 ± 0.01a
ICC-US-10 min-S	2.01 ± 0.03ab	0.41 ± 0.01ab
ICC-US-20 min-S	2.24 ± 0.05d	0.41 ± 0.01ab
ICC-US-40 min-S	2.14 ± 0.08 cd	0.42 ± 0.01b
CS-US-ICC-S	2.09 ± 0.03bc	0.40 ± 0.00ab

1 All data are averages of triplicate measurements with standard deviation. Means in a column with different letters are significantly different (p < 0.05) by the least significant difference (Tukey) test.

2 ICC-S, starch isolated from intact chickpea cells; ICC-US-10 min-S, starch isolated from intact chickpea cells treated with 10 min ultrasound; ICC-US-20 min-S, starch isolated from intact chickpea cells treated with 20 min ultrasound; ICC-US-40 min-S, starch isolated from intact chickpea cells treated with 40 min ultrasound; CS-US-ICC-S, starch isolated from intact chickpea cells obtained from ultrasound treated chickpea seeds.

#### 3.2.3 Thermal properties of isolated starch

The thermal properties of isolated starch (ICC-S, CS-US-ICC-S, ICC-US-10 min-S, ICC-US-20 min-S, ICC-US-40 min-S, Fig. 1) are shown in Table 2. The results indicate no significant differences in the thermal properties between the starch isolated from ultrasound-treated and untreated intact chickpea cells. In many previous researches, the gelatinization temperature was reported to be unaffected after ultrasonication [4], [6], [38]. However, decreased *ΔH* was observed in many studies with the increase of ultrasound time, due to the energy of cavitation effects disrupting starch amorphous regions, and damaging the surface of starch, which leads to enhancement of water penetration to the crystalline regions of starch [5], [6], [38], [39], [40]. In the present research, no significant difference was observed after the starch was isolated from ultrasound-treated intact cells, possibly due to the cell wall shielding the inner starch from damage by the ultrasound power. Despite the FT-IR and XRD data suggesting that the crystalline structure of starch may undergo subtle changes after ultrasound treatment, these changes are not significant enough to cause measurable alterations in the thermal properties of starch.

**Table 2:** Thermal properties of ultrasound treated chickpea starch isolates.^1^

Samples^2^	*T_o_*	*T_p_*	*T_c_*	Δ*H*
ICC-S	67.75 ± 0.96a	75.86 ± 0.04a	86.13 ± 1.10a	7.30 ± 0.06a
ICC-US-10 min-S	67.88 ± 0.12a	75.69 ± 0.09a	85.66 ± 0.42a	7.41 ± 0.29a
ICC-US-20 min-S	68.7 ± 0.581a	76.04 ± 1.60a	86.97 ± 1.30a	7.41 ± 0.25a
ICC-US-40 min-S	67.70 ± 0.54a	75.53 ± 0.35a	86.04 ± 1.54a	7.29 ± 0.35a
CS-US-ICC-S	68.46 ± 0.33a	75.92 ± 0.15a	84.53 ± 1.69a	7.43 ± 0.19a

1 All data are averages of triplicate measurements with standard deviation. Means in a column with different letters are significantly different (p < 0.05) by the least significant difference (Tukey) test.

2 ICC-S, starch isolated from intact chickpea cells; ICC-US-10 min-S, starch isolated from intact chickpea cells treated with 10 min ultrasound; ICC-US-20 min-S, starch isolated from intact chickpea cells treated with 20 min ultrasound; ICC-US-40 min-S, starch isolated from intact chickpea cells treated with 40 min ultrasound; CS-US-ICC-S, starch isolated from intact chickpea cells obtained from ultrasound treated chickpea seeds.

### 3.3 Effects of ultrasound treatment on the structural characteristics of protein

To investigate the effects of ultrasound treatment on the structural characteristics of protein inside of intact chickpea cells, the protein was isolated from all the cells samples, and the thermal properties, FT-IR spectrum, and SDS-PAGE were studied.

#### 3.3.1 Thermal properties

The thermal properties of isolated protein (ICC-P, CS-US-ICC-P, ICC-US-10 min-P, ICC-US-20 min-P, ICC-US-40 min-P, Fig. 1), as shown in Table 3, reveal that ultrasound treatment does not significantly affect the thermal stability of proteins within the cell walls, with the exception of a slight increase in peak temperature correlating with ultrasound duration. Contrary to other studies on ultrasound-treated proteins, which reported changes in denaturation temperature and a decrease in enthalpy post-sonication [11], [12], [13], the results demonstrate that the *ΔH* remained stable across all samples. Theoretically, when protein isolates suspension treated with high-intensity ultrasound, due to the cavitation effects of ultrasound, the regional elevation of temperature and pressure at the point of bubble collapse, this condition can disrupt the non-covalent bonds within protein molecules, such as hydrogen bonds, hydrophobic interactions or break the covalent bonds in protein molecules, like disulfide bonds, therefore causing protein denaturation [11], [41]. The stable thermal properties of protein isolated from ultrasound-treated intact chickpea cells suggest that the structure of the cell wall acts as a protective barrier, shielding the entrapped proteins from ultrasound-induced denaturation.

**Table 3:** Thermal properties of ultrasound treated chickpea protein isolates.^1^

Samples^2^	*To*	*Tp*	*Tc*	Δ*H*
ICC-P	74.76 ± 1.66a	87.69 ± 0.06a	99.03 ± 1.99a	8.02 ± 0.21a
ICC-US-10 min- P	74.34 ± 0.05a	88.66 ± 0.19bc	101.03 ± 2.25a	8.00 ± 0.15a
ICC-US-20 min- P	73.63 ± 1.07a	88.66 ± 0.25bc	100.21 ± 1.63a	8.14 ± 0.36a
ICC-US-40 min- P	73.10 ± 0.62a	89.38 ± 0.34c	100.50 ± 0.30a	8.08 ± 0.15a
CS-US-ICC- P	73.21 ± 0.70a	87.86 ± 0.44ab	99.74 ± 1.35a	8.15 ± 0.16a

1 All data are averages of triplicate measurements with standard deviation. Means in a column with different letters are significantly different (p < 0.05) by the least significant difference (Tukey) test.

2 ICC-P, protein isolated from intact chickpea cells; ICC-US-10 min-P, protein isolated from intact chickpea cells treated with 10 min ultrasound; ICC-US-20 min-P, protein isolated from intact chickpea cells treated with 20 min ultrasound; ICC-US-40 min-P, protein isolated from intact chickpea cells treated with 40 min ultrasound; CS-US-ICC-P, protein isolated from intact chickpea cells obtained from ultrasound treated chickpea seeds.

#### 3.3.2 Secondary structure of isolated protein

**Fig. 5:**
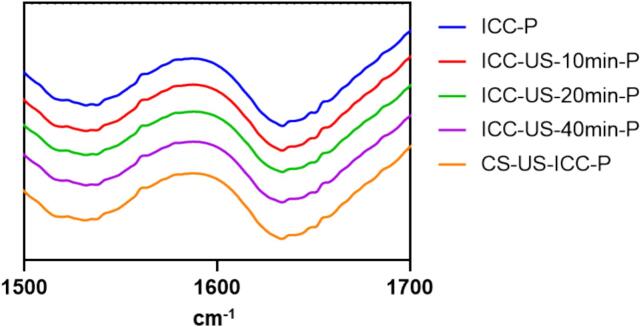
FT-IR spectra of ultrasound treated chickpea protein isolates (ICC-P, protein isolated from intact chickpea cells; ICC-US-10 min-P, protein isolated from intact chickpea cells treated with 10 min ultrasound; ICC-US-20 min-P, protein isolated from intact chickpea cells treated with 20 min ultrasound; ICC-US-40 min-P, protein isolated from intact chickpea cells treated with 40 min ultrasound; CS-US-ICC-P, protein isolated from intact chickpea cells obtained from ultrasound treated chickpea seeds.).

FT-IR spectroscopy was used to study the secondary structure of protein. Fig. 5 illustrates the FT-IR spectra of isolated chickpea protein following different durations of ultrasound treatment. All protein isolates (ICC-P, CS-US-ICC-P, ICC-US-10 min-P, ICC-US-20 min-P, ICC-US-40 min-P, Fig. 1) show characteristic bands corresponding to the C = O stretching vibration of the peptide backbone of proteins at region of 1600–1700 cm^−1^ (amide I), N-H bending vibrations and C-N stretching vibrations at region of 1500–1600 cm^−1^ (amide II), and vibrations involving the C-N stretch and N-H bend at region of 1200–1300 cm^−1^ (amide III) (Haris & Severcan, 1999). The spectra of all samples were similar, with variations in absorbance intensity observed.

Since the Amide I region is sensitive to the protein secondary structure, the peak position of amide I was analysed to estimate the secondary structure of protein isolates. The content of different secondary structures is shown in Table 4. The results indicated a slight decrease in β-sheet content and a small increase in α-helix content as the duration of ultrasound treatment increased. The content of random coil and β-turn remained consistent before and after ultrasound treatment. The increase in α-helix content and decrease in β-sheet structures post-ultrasound treatment align with findings from previous studies [9], [41], [42], [43]. This alteration may be attributed to the ultrasonic energy inducing conformational changes in the protein, promoting the formation of α-helices, which are generally more flexible and less tightly packed than β-sheets. This structural rearrangement might lead to greater exposure of peptide bonds and affect the protein functional properties including enzymatic activity.

**Table 4:** Secondary structure contents of ultrasound-treated chickpea cells protein isolates.^1^

Samples^2^	β-sheet	random coil	α-helix	β-turn
ICC-P	39.28 ± 0.22a	15.02 ± 0.09a	25.43 ± 0.29a	20.27 ± 0.17a
ICC-US-10 min- P	38.17 ± 0.26b	15.03 ± 0.10a	25.98 ± 0.10ab	20.81 ± 0.26a
ICC-US-20 min- P	38.18 ± 0.35b	14.82 ± 0.02a	26.14 ± 0.22b	20.87 ± 0.11a
ICC-US-40 min- P	38.44 ± 0.13ab	14.86 ± 0.01a	26.06 ± 0.09ab	20.64 ± 0.04a
CS-US-ICC- P	38.22 ± 0.19b	15.04 ± 0.10a	26.02 ± 0.05ab	20.73 ± 0.14a

1 All data are averages of triplicate measurements with standard deviation. Means in a column with different letters are significantly different (p < 0.05) by the least significant difference (Tukey) test.

2 ICC-P, protein isolated from intact chickpea cells; ICC-US-10 min-P, protein isolated from intact chickpea cells treated with 10 min ultrasound; ICC-US-20 min-P, protein isolated from intact chickpea cells treated with 20 min ultrasound; ICC-US-40 min-P, protein isolated from intact chickpea cells treated with 40 min ultrasound; CS-US-ICC-P, protein isolated from intact chickpea cells obtained from ultrasound treated chickpea seeds.

#### 3.3.3 SDS-PAGE

**Fig. 6:**
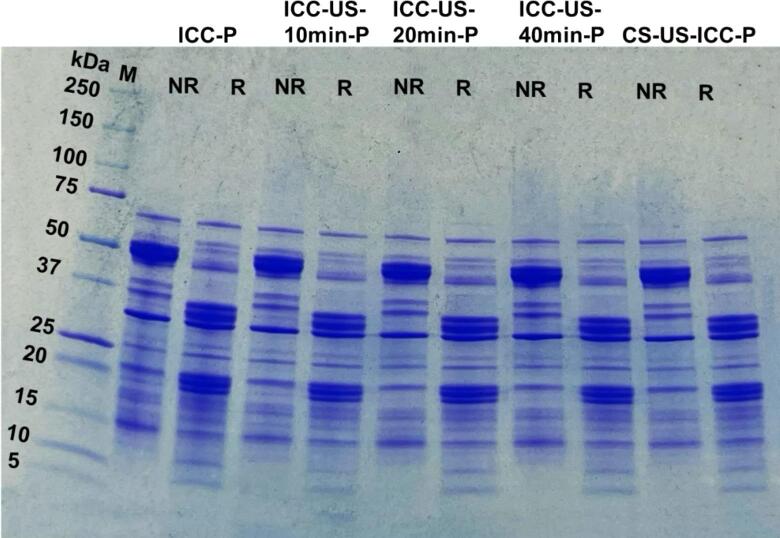
SDS-PAGE patterns of ultrasound treated chickpea cells protein isolates under non-reducing (NR) and reducing conditions (R) (ICC-P, protein isolated from intact chickpea cells; ICC-US-10 min-P, protein isolated from intact chickpea cells treated with 10 min ultrasound; ICC-US-20 min-P, protein isolated from intact chickpea cells treated with 20 min ultrasound; ICC-US-40 min-P, protein isolated from intact chickpea cells treated with 40 min ultrasound; CS-US-ICC-P, protein isolated from intact chickpea cells obtained from ultrasound treated chickpea seeds.).

The SDS-PAGE was used to identify the major protein constituents of all isolated protein samples. Fig. 6 displays that both the untreated and ultrasound-treated samples exhibit similar protein electrophoretic patterns, indicating that ultrasound treatment does not alter the molecular size and confirmation of the protein. The result is supported by the findings of Hu et. al. (2021), Jiang et. al. (2014) and Hu et. al. （2013）, who reported that the molecular weight of non-thermally sonicated soybean and black bean isolates remained unchanged even after treatment under conditions of 600 W for 30 min [8], [9], [10].

Based on these results, it can be concluded that ultrasound treatment does not significantly impact the structure of starch and protein, the notable difference of starch/protein digestibility is not related to the alteration of micro-level of structure of starch and protein, but is due to the alterations in the porosity of cell wall structure, allowing the easy access of enzymes towards starch and protein for hydrolysis,

### 3.4 Effects of ultrasound treatment on the cell wall structure

To investigate the impact of ultrasound treatment on the cell wall degradation, the monosaccharide composition of the cell walls isolated from the intact cells before and after sonification was analysed. The surface morphology was observed with SEM, the permeability of cell wall was detected with the FITC dextran to mimic the digestive enzymes.

#### 3.4.1 Monosaccharide composition of cell wall

**Fig. 7:**
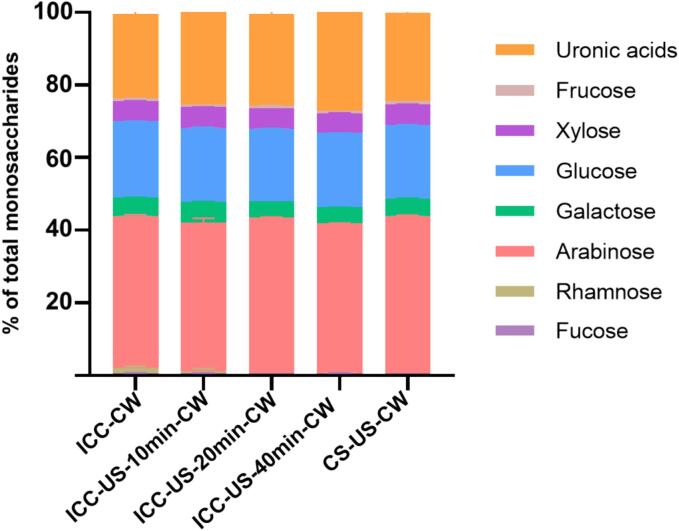
Monosaccharides composition of cell walls isolated from ultrasound treated chickpea cells (ICC-CW, cell wall isolated from intact chickpea cells; ICC-US-10 min-CW, cell wall isolated from intact chickpea cells treated with 10 min ultrasound; ICC-US-20 min-CW, cell wall isolated from intact chickpea cells treated with 20 min ultrasound; ICC-US-40 min-CW, cell wall isolated from intact chickpea cells treated with 40 min ultrasound; CS-US-ICC-CW, cell wall isolated from intact chickpea cells obtained from ultrasound treated chickpea seeds.).

In the current study, the cell walls were isolated from all chickpea cell samples (ICC-CW, CS-US-ICC-CW, ICC-US-10 min-CW, ICC-US-20 min-CW, ICC-US-40 min-CW, Fig. 1) and their monosaccharide composition is listed in Fig. 7. Aligning with prior research, the chickpea cell wall, a type II cell wall mainly composed of arabinose, glucose and uronic acids, but also includes xylose, galactose, rhamnose, fructose and fucose [44]. The results for neutral sugar content show that both ultrasound-treated and untreated samples had similar monosaccharide profiles, corroborating findings from previous studies which indicated that ultrasound treatment at 20 Hz does not alter the monosaccharide composition of plant polysaccharides, even after 10 h of exposure [45], [46], [47], [48]. In terms of uronic acid content which is the primary component of pectin, a notable increase was observed with the prolongation of ultrasound treatment time. This proves that the pectin composition of cell walls is more susceptible to ultrasound than cellulose and hemicellulose, as compared with the cellulose and hemicellulose which have more liner structure providing stronger intermolecular interactions [49], [50], the high-branched pectin has a less rigid structure (Pancerz, Kruk, & Ptaszek, 2022). This finding aligns with outcomes seen in ultrasound-treated sugar beet, *Premna microphylla*, *Turcz Flammulina velutipes* and apple polysaccharides [51], [52], [53], [54]. In these studies, it was revealed that the pectin backbone, primarily composed of uronic acid, demonstrates enhanced stability compared to its side chains when exposed to ultrasonic waves, thereby resulting in an increased uronic acid content in polysaccharide subjected to ultrasound treatment. This phenomenon can be attributed to the intrinsic structural resilience of uronic acid, which makes it less prone to ultrasonic degradation. The enhanced stability of the uronic acid-rich backbone under ultrasound treatment indicates a selective degradation mechanism, wherein ultrasonic energy preferentially targets and breaks down more vulnerable molecular bonds found in side-chains rather than the uronic acid-rich backbones. This selective degradation not only preserves but potentially concentrates uronic acid within the cell wall matrix, enriching its composition.

#### 3.4.2 Microscopic observations of cell wall

**Fig. 8:**
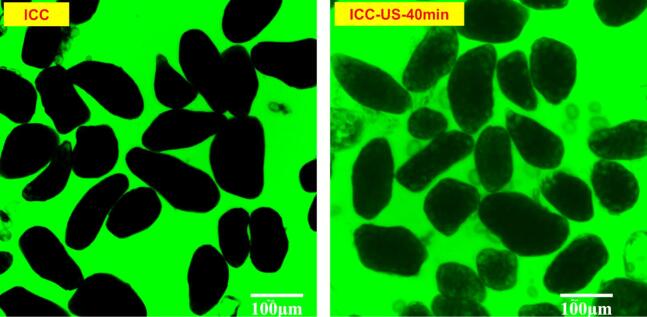
CLSM micrographs of intact chickpea cells (ICC, left) and intact chickpea cells ultrasound treated for 40 min (ICC-US-40 min, right) suspended in FITC-dextran solution.

In order to visualise the permeability of chickpea cell walls before and after 40 min ultrasound, 70 kDa FITC-dextran probe which has similar size with digestive enzymes was used to identify the permeability of the cell structure to digestive enzymes [28], [55], [56]. As shown in Fig. 8, negligible fluorescence was detected within the intact chickpea cells before ultrasound treatment, confirming that the intact cell wall can provide a strong physical barrier that blocks digestive enzymes from accessing the internal nutrients. Conversely, noticeable fluorescence was observed inside the intact chickpea cells after 40 min ultrasound treatment, demonstrating increased permeability. Moreover, despite most cells remaining intact, some starch granules were seen scattered outside of intact cells along with empty cell walls, implying that ultrasonic waves can partially degrade the cell wall, thereby enhancing its permeability. These results clarify why the digestibility of non-treated intact chickpea cells is initially low but tends to improve as the ultrasound treatment time increases [19], [28].

**Fig. 9:**
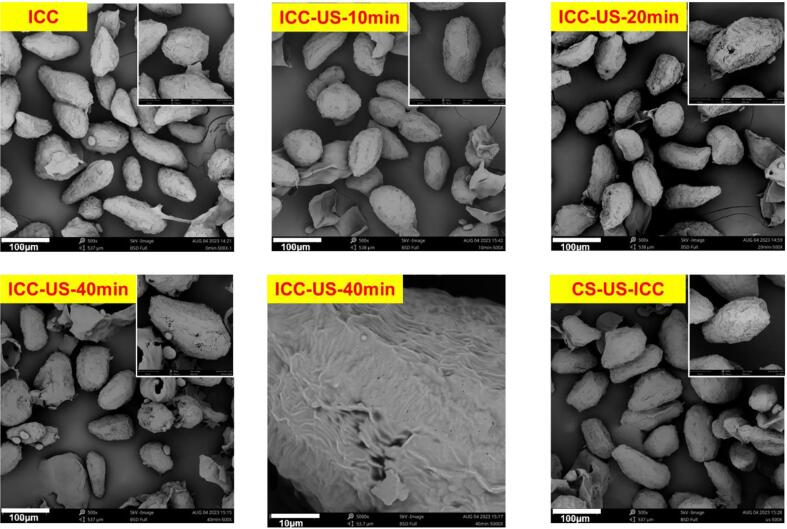
Scanning electron micrographs of ultrasound treated chickpea cell (ICC, untreated intact chickpea cells; ICC-US-10 min, intact chickpea cells treated with 10 min ultrasound; ICC-US-20 min, intact chickpea cells treated with 20 min ultrasound; ICC-US-40 min, intact chickpea cells treated with 40 min ultrasound; CS-US-ICC, intact chickpea cells isolated from ultrasound treated chickpea seeds.).

To visualise the destruction of cell walls by ultrasound, the surface structure of cell samples (ICC, CS-US-ICC, ICC-US-10 min, ICC-US-20 min, ICC-US-40 min, Fig. 1) was observed with SEM and shows in Fig. 9. From the results, it was known that all the cells samples kept relative integrity, even after 40 min ultrasonification. The starch granules and protein matrix are tightly packed in the cell wall, the density structure of the macronutrients protect them from being damaged by the energy of ultrasound compared with the starch and/or protein suspension, and may explain the fact that the physiochemical properties of starch and protein that isolated from the ultrasound treated intact chickpea cells did not be significantly changed compared with the starch and protein isolated from the untreated intact cells. Targeting on the cells surface morphology, it is observed that the ICC sample which was not treated with ultrasound, displayed cells with intact, wrinkled surfaces and regular shapes. The cells isolated from 40 min ultrasound treated chickpea seeds (CS-US-ICC) look similar with non-treated intact chickpea cells, indicating that ultrasound treatment of the whole chickpea seed did not damage its cellular macrostructure. After 10 min of ultrasound treatment on isolated chickpea cells (ICC-US-10 min), minor surface changes were observed, yet most of the particles retained their integrity. Samples subjected to 20 min of ultrasound treatment (ICC-US-20 min) exhibited more noticeable surface damage, with some particles starting to exhibit cracks and breakdown on the surface. With ultrasound treatment extending to 40 min (ICC-US-40 min), the cell destruction is more significant, surfaces show extensive indentations and cracks, indicating severe damage to the cell walls. In addition to the visible cracks, some micropores were also noticeable in the enlarged images.

The result is consistent with many previous researches showed that the ultrasound treatment can disrupt the plant cell walls via the sponge effect and the cavitation effect produced by acoustic waves [57], [58], [59], [60]. As evident by our findings, when ultrasound waves interact with intact cells, plant cell walls are the primary surface of contact. The acoustic bubbles developing in the close proximity of cell walls might get affected by them and develop an asymmetrical shape, which upon rupturing might create acoustic jets driven towards the cell walls. These jets along with the high localised pressure and the temperatures could prompt the degradation of larger cell wall components like cellulose, hemicellulose and pectin, thus resulting in surface erosion and cavity formation with the cell wall structure [61], [62], and with increase of porosity of cell walls, the digestion rate and extent of inner macronutrients is going up with the ultrasound treatment time.

## 4 Conclusion

The present study investigates the ultrasound effect on different levels of cellular organisations, namely whole chickpeas and isolated intact cells. We found that the effectiveness of ultrasound treatment varies significantly with proximity to the surfaces in direct contact with the sonication media. Whole beans were least susceptible to ultrasonic effects compared to isolated intact chickpea cells, primarily due to shielding/cushion effects. For isolated intact chickpea cells, increased ultrasound treatment time translated into improved digestibility of entrapped starch and proteins, indicating that ultrasound can modulate the accessibility of digestive enzymes to entrapped macromolecules within the cell walls by physically damaging them. However, structural characterizations of starch and protein suggested that ultrasound did not significantly alter the molecular structures of these macromolecules. This further elucidates that the increased digestibility is not a result of micro-level physicochemical alterations of molecules but of the cell walls. Confocal laser scanning microscopy (CLSM) and scanning electron microscopy (SEM) observations confirmed that ultrasound treatment could cause noticeable changes to the cell wall's surface, leading to increased permeability. SEM images showed progressive surface damage with extended ultrasound treatment time, with pronounced cracks found after 40 min of treatment, supporting the hypothesis that ultrasound treatment increase the macronutrients digestibility by breaking the cell wall structure, which is a natural physical barrier. In summary, ultrasound treatment has the potential to enhance the digestibility of macromolecules in legumes by altering the integrity of the cell wall rather than by changing the intrinsic properties of the macromolecules themselves. This could pave the way for the development of novel food processing strategies.

## CRediT authorship contribution statement

**Weiyan Xiong:** Writing – original draft, Validation, Software, Methodology, Investigation, Formal analysis, Data curation, Conceptualization. **Gaurav Kumar:** Writing – review & editing. **Bin Zhang:** Writing – review & editing, Supervision, Resources. **Sushil Dhital:** Writing – review & editing, Supervision, Resources.

## Funding statement

This research was funded by the Australian Government through Australian Research Council 333 (ARC) Linkage Project (LP210200616).

## Declaration of competing interest

The authors declare that they have no known competing financial interests or personal relationships that could have appeared to influence the work reported in this paper.
